# Preparation Optimization and Antioxidant Properties of the *β*-Glucan and Ferulic Acid/Quercetin Complex from Highland Barley (*Hordeum vulgare* var. *nudum*)

**DOI:** 10.3390/foods14152712

**Published:** 2025-08-01

**Authors:** Yuanhang Ren, Yanting Yang, Mi Jiang, Wentao Gu, Yanan Cao, Liang Zou, Lianxin Peng

**Affiliations:** 1 Key Laboratory of Coarse Cereal Processing, Ministry of Agriculture and Rural Affairs, Sichuan Engineering and Technology Research Center of Coarse Cereal Industralization, College of Food and Biological Engineering, Chengdu University, Chengdu 610106, China; 2 Agricultural Science Research Institute of Tibetan Autonomous Prefecture of Ganzi Prefecture, Kangding 626099, China

**Keywords:** highland barley, ferulic acid, quercetin, *β*-glucan, phenol–polysaccharide complex, antioxidation

## Abstract

Polysaccharides and phenols are commonly co-localized in various plant-derived foods, including highland barley (*Hordeum vulgare* L. var. *nudum* Hook. f.). The interactions between these compounds can influence multiple characteristics of food products, including their physicochemical properties and functional performance, such as bioavailability, stability, and digestibility, which may support promising application of the phenol and polysaccharide complex in health food industry. In this study, two complexes with potential existence in highland barley, such as *β*-glucan-ferulic acid (GF) and *β*-glucan-quercetin (GQ), were prepared using the equilibrium dialysis method in vitro. FTIR and SEM results showed that ferulic acid and quercetin formed complexes with *β*-glucan separately, with covalent and non-covalent bonds and a dense morphological structure. The pH value, reaction temperature, and concentration of phosphate buffer solution (PBS) were confirmed to have an impact on the formation and yield of the complex. Through the test of the response surface, it was found that the optimum conditions for GF and (GQ) preparations were a pH of 6.5 (6), a PBS buffer concentration of 0.08 mol/L (0.3 mol/L), and a temperature of 8 °C (20 °C). Through in vitro assays, GF and GQ were found to possess good antioxidant activity, with a greater scavenging effect of DPPH, ABTS, and hydroxyl radical than the individual phenolic acids and glucans, as well as their physical mixtures. Taking GF as an example, the DPPH radical scavenging capacity ranked as GF (71.74%) > ferulic acid (49.50%) > PGF (44.43%) > *β*-glucan (43.84%). Similar trends were observed for ABTS radical scavenging (GF: 54.56%; ferulic acid: 44.37%; PGF: 44.95%; *β*-glucan: 36.42%) and hydroxyl radical elimination (GF: 39.16%; ferulic acid: 33.06%; PGF: 35.51%; *β*-glucan: 35.47%). In conclusion, the convenient preparation method and excellent antioxidant effect of the phenol–polysaccharide complexes from highland barley provide new opportunities for industrial-scale production, development, and design of healthy food based on these complexes.

## 1. Introduction

Phenols (important secondary metabolites) and polysaccharides (structural cell wall components) are both widely present in grains, fruits, vegetables, and various other foods [1,2]. Natural phenol and polysaccharide possess many activities, such as antitumor, antioxidant, anti-inflammatory, and anti-obesity effects [3,4]. Several studies have confirmed that phenol and polysaccharide in plant-derived foods and their processed products can form complexes through covalent and non-covalent interactions [5,6,7]. These molecular interactions of phenol–polysaccharide can significantly modify key food properties, including digestibility, nutrient bioavailability, product stability, and physiological functionality, compared to their individual components [8,9]. As a result, these phenol–polysaccharide complexes (PPC) have gained significant research interest due to their promising potential in functional food applications.

Highland barley (*Hordeum vulgare* L. var. *nudum* Hook. f.) is an important crop of the Qinghai Tibet Plateau region with a wide planting history. As the main edible part, barley grains are rich in various bioactive ingredients, such as polysaccharide, phenol, dietary fiber, etc., exhibiting potential nutritional value and health benefits [10,11]. *β*-glucan, as a representative substance of polysaccharide in barley, mainly exists in the endosperm layer and the endosperm cell wall, and it has been proven to have good hypolipidemic and antioxidant properties [12]. Meanwhile, phenol of highland barley, including ferulic acid, gallic acid, and quercetin, is also an important component in the outer layer of barley seeds [13], particular colored barley, with bioactive constituents possessing various health benefits, such as antioxidant, anti-inflammatory, and anti-obesity effects [14]. Notably, phenol often exists in both free and bound forms in nature or food processing. Due to the co-localization of phenol and polysaccharide in highland barley, it serves as an ideal model for studying phenol–polysaccharide interactions and provides a novel direction for the improvement of functional barley products. However, there is still limited research on the structural characteristics, binding mechanisms, synergistic biological functions, and, in particular, convenient in vitro preparation methods of PPC in highland barley [8,9].

In order to verify the possibility of combining *β*-glucan and phenols in barley bran under natural conditions, as well as the potential health benefits brought by the PPC, in this study, two complexes with potential existence in highland barley, such as *β*-glucan-ferulic acid (GF) and *β*-glucan-quercetin (GQ), were prepared in vitro through the equilibrium dialysis method instead of chemical grafting for the first time. Then, the binding mode and morphological characteristics of the complexes were further studied through FTIR and SEM assay, which confirmed that ferulic acid and quercetin formed complexes with *β*-glucan separately, with covalent bonds and a dense morphological structure. The effects of different pH values, PBS concentrations, and temperatures on the adsorption capacity of complexes were also investigated to find an optimal preparation condition for GF and GQ using the equilibrium dialysis method. Through the test of the response surface, the optimum conditions for GF (GQ) preparation were a pH of 6.5 (6), a PBS buffer concentration of 0.08 mol/L (0.3 mol/L), and a temperature of 8 °C (20 °C). In addition, the free radical (DPPH, ABTS, and hydroxyl radical) scavenging effect showed that the GF and GQ had good in vitro antioxidant activity, which further expands the current understanding of PPC bioactivity. In summary, the convenient and large-scale preparation method and excellent antioxidant effect of the PPC from highland barley open new opportunities for the exploitation of the industrial production, development, and design of healthy food based on these complexes.

## 2. Materials and Methods

### 2.1. Materials

*β*-glucan (purity ≥ 95.0%, Art. No. S52919), ferulic acid (purity ≥ 99.8%, Art. No. A10353), and quercetin (purity ≥ 98.0%, Art. No. B20527) standards were purchased from Yuanye Biotechnology Co., Ltd. (Shanghai, China). An MD44-14 (MW: 8000–14000) dialysis bag was purchased from Union Carbide (Danbury, CT, USA). DPPH, ABTS, and hydroxyl free radical scavenging capacity assay kit were purchased from Beijing Solebao Biotechnology Co., Ltd. (Beijing, China). Phosphate buffer solution (PBS) and other reagents used in this research were obtained from Kelong Chemical Reagent Co., Ltd. (Chengdu, China).

### 2.2. Sample Pretreatment

*β*-glucan: *β*-glucan was mixed with deionized water and stirred at 80 °C until complete dissolution. The above dissolved *β*-glucan was put into a dialysis bag. The bag was dialyzed with running water for 24 h and then dialyzed with distilled water for another 24 h to remove small molecule compounds and avoid their interference with adsorption capacity calculations. At last, the pre-treated *β*-glucan was prepared to a concentration of 0.5 mg/mL of solution and stored at 4 °C for later use.

Dialysis bag: Dialysis bags were trimmed to 10 cm segments and subjected to a 10 min boil in a pretreatment solution containing 1 mmol/L of EDTA and 2% (*w*/*v*) sodium bicarbonate. Following extensive rinsing with deionized water, each bag was transferred to a beaker and fully immersed in deionized water. The system was then covered with tin foil and sterilized via moist heat treatment (10 min). Processed bags were stored at 4 °C in deionized water to maintain complete submersion until use.

### 2.3. Equilibrium Dialysis Assay

The 6 mL aliquot of pretreated *β*-glucan solution (0.5 mg/mL) was combined with 2 mL of either ferulic acid or quercetin solution (both at 0.5 mg/mL). The resulting mixture was transferred to dialysis bags and subjected to sequential dialysis, first against 30 mL of PBS buffer in covered 50 mL centrifuge tubes for 16 h until equilibrium was attained, as determined by stable absorbance measurements (λ = 273 nm) of the external solution. The dialysate concentrations of ferulic acid and quercetin were quantified through UV spectrophotometry using their respective standard curves (ferulic acid: Y = 21.525X + 0.2366; quercetin: Y = 25.746X + 0.0232). The adsorption capacity of *β*-glucan for each polyphenol was subsequently calculated using the following equation [15,16]:

$$\mathrm{Adsorption}\mathrm{Capacity}\;(ug/mg)=\frac{M_{tp}-C_{t}V}{M}$$

$M_{tp}$ represents the initial total weight of phenol (µg), $C_{t}$ represents the concentration of phenol in the external dialysis solution at equilibrium (µg/mL), V represents the total volume of the internal and external dialysis solutions (mL), and M represents the total amount of *β*-glucan (mg).

### 2.4. FTIR Spectroscopy

Following Meng et al.’s protocol [17], we prepared samples by homogenizing 1 mg of PPC with 120 mg of KBr, followed by pellet formation and FTIR analysis (Bruker Optics, Ettlingen, Germany) across the 4000–400 cm^−1^ spectral range under the following conditions: sample scanning times: 32; background scanning times: 32; resolution: 4.000; sampling gain: 1.0; moving mirror speed: 0.4747; aperture: 100.0.

### 2.5. Scanning Electron Microscope

The dried samples was adhered to a conductive carbon film with double-sided adhesive, followed by placement in the sample chamber of an ion sputtering instrument. A gold coating was applied under conditions of 10 kV for 60 s. The above samples were scanned and photographed under high vacuum conditions with an acceleration voltage of 30 kV and a magnification of 500 and 5000 times [18].

### 2.6. Antioxidant Activity

For the DPPH assay, 975 µL of DPPH solution was mixed with 25 µL of aqueous solution of *β*-glucan, ferulic acid, quercetin, physically mixed *β*-glucan and ferulic acid (PGF), physically mixed *β*-glucan and quercetin (PGQ), GF, and GQ at a concentration of 5 mg/mL. The absorbance at 515 nm occurred at room temperature for 30 min on a plate reader (Biotek Powerwave XS, Santa Clara, CA, USA). Results were expressed as the percentage of the scavenging rate of 0.3 mg/mL of vitamin C equivalents [19]. The calculation formula references the manufacturer’s instructions: DPPH scavenging rate = [1 − (A1 − A2)/A3] × 100%.

For the ABTS assay, 850 µL of ABTS solution was mixed with 100 µL of H_2_O_2_ and 50 µL of aqueous solution of *β*-glucan, ferulic acid, quercetin, physically mixed *β*-glucan and ferulic acid (PGF), physically mixed *β*-glucan and quercetin (PGQ), GF, and GQ at a concentration of 5 mg/mL. The absorbance at 405 nm occurred at room temperature for 6 min on a plate reader. Results were expressed as the percentage of the scavenging rate of 1 mmol/L of vitamin C equivalents [20]. The calculation formula references the manufacturer’s instructions: ABTS scavenging rate = [1 − (Aa − Ab)/Ac] × 100%.

For the hydroxyl radical scavenging assay, 80 µL of hydroxyl radical solution was mixed with 160 µL of 4-aminobenzenesulfonamide and 80 µL of aqueous solution of *β*-glucan, ferulic acid, quercetin, physically mixed *β*-glucan and ferulic acid (PGF), physically mixed *β*-glucan and quercetin (PGQ), GF, and GQ at a concentration of 5 mg/mL. The absorbance at 550 nm occurred at room temperature for 1 min after the above mixture was evenly mixed with the N-(1-Naphthyl)-Ethylenediamine on a plate reader. Results were expressed as the percentage of the scavenging rate of 2 mg/mL of vitamin C equivalents [21]. The calculation formula references the manufacturer’s instructions: hydroxyl radical scavenging rate = [Ao − (Am − An)/Ao] × 100%.

### 2.7. Optimization of the Preparation Process of GB and GQ

One-way experiment: The effects of three factors, including pH, PBS concentration, and temperature, on the preparation of GF (GQ) were investigated using the adsorption capacity as the indicator.

Response surface experimental design: Box–Behnken (Version 7.0) was used to design a three-factor, three-level response surface test, and the experimental design is shown in Table 1 and Table 2.

### 2.8. Statistics and Analysis of Data

Statistical analyses were conducted in GraphPad Prism 9.5.0 (GraphPad Software, San Diego, CA, USA). Data were analyzed through ANOVA followed by Duncan’s post hoc test (*p* < 0.05) to determine significant differences among treatment groups. All experiments were repeated at least three times, and the data were expressed as mean ± standard error.

## 3. Results

### 3.1. Characterization of GF and GQ

**Figure 1:**
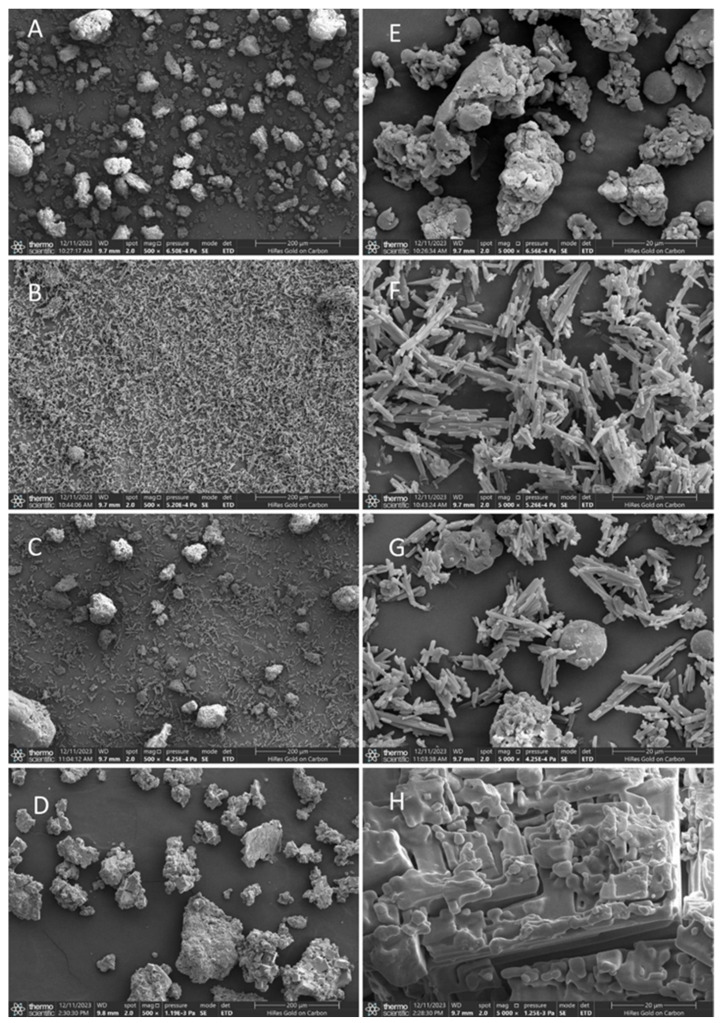
SEM images of *β*-glucan–ferulic acid complexes (GF). Submicroscopic structure of individual *β*-glucan (**A**), individual ferulic acid (**B**), physical mixture of *β*-glucan–ferulic acid (**C**), and GF (**D**) at 500× magnification. Submicroscopic structure of individual *β*-glucan (**E**), individual ferulic acid (**F**), physical mixture of *β*-glucan–ferulic acid (**G**), and GF (**H**) at 5000× magnification.

**Figure 2:**
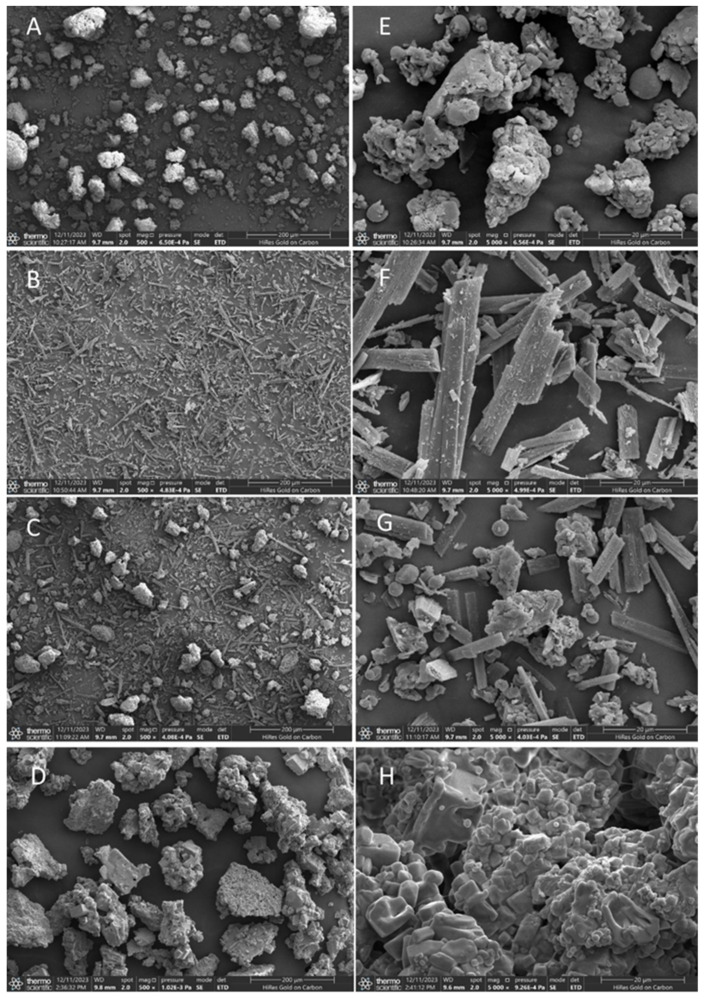
SEM images of *β*-glucan–quercetin complexes (GQ). Submicroscopic structure of individual *β*-glucan (**A**), individual quercetin (**B**), physical mixture of *β*-glucan–quercetin (**C**), and GQ (**D**) at 500× magnification. Submicroscopic structure of individual *β*-glucan (**E**), individual quercetin (**F**), physical mixture of *β*-glucan–quercetin (**G**), and GQ (**H**) at 5000× magnification.

As shown in the SEM observation results, the *β*-glucan and two phenols exhibit significant differences in monomeric morphology under an ultrafine structure. The individual *β*-glucan presents an amorphous block and sheet structures with a particle size of approximately 50 μm (Figure 1A and Figure 2A). Free ferulic acid (Figure 1B) and quercetin (Figure 2B) exist in a needle-like crystal form with lengths between 20 and 40 μm. Furthermore, Figure 1C and Figure 2C show that the physical mixing of polysaccharide and phenol did not alter their respective morphological structures. However, the substance prepared using the equilibrium dialysis method exhibits a microstructure different from that of *β*-glucan and phenol alone. These substances have larger particle sizes between approximately 50 and 200 μm. The surface of the substances shown in Figure 1D and Figure 2D became denser and rougher compared to individual *β*-glucan, which may be attributed to the hydrophobic aromatic rings of ferulic acid and quercetin embedded in the polysaccharide network structure, forming a more compact structure. In addition, the needle-shaped free phenol crystals were not found in the figures. The above results confirm that under equilibrium dialysis assay, *β*-glucan could bind ferulic acid and quercetin, respectively, and form phenol–polysaccharide complexes GF and GQ.

**Figure 3:**
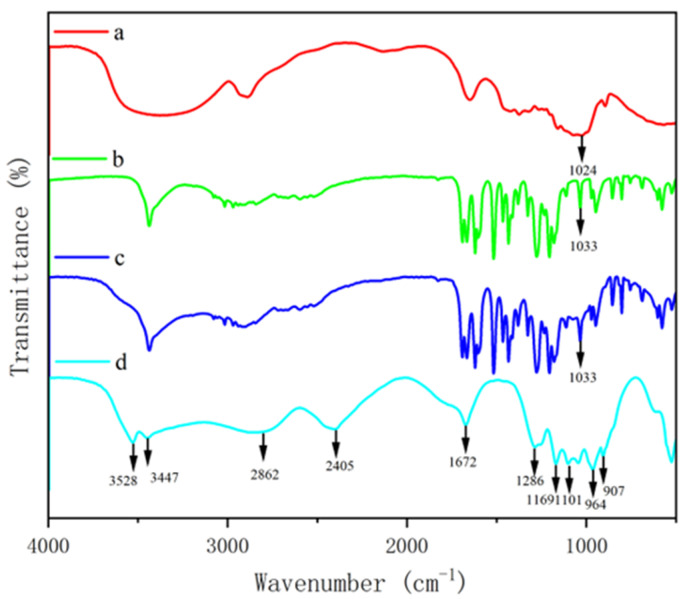
FTIR spectra of *β*-glucan and ferulic acid complex. *β*-glucan (a), ferulic acid (b), PGF (c), GF (d).

**Figure 4:**
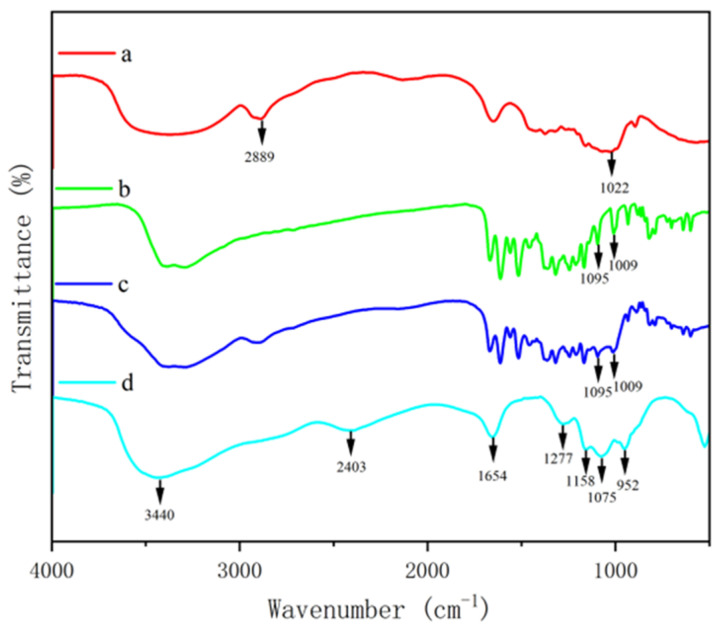
FTIR spectra of *β*-glucan and quercetin complex. *β*-glucan (a), quercetin (b), PGQ (c), GQ (d).

To further confirm the chemical crosslinking between polysaccharide and phenol, FTIR was used to detect the absorption peaks of GF and GQ. Figure 3 and Figure 4 show the changes in the FTIR spectra of *β*-glucan, ferulic acid (quercetin), physically mixed *β*-glucan and ferulic acid (quercetin), PGF (PGQ), and GF (GQ). These changes can be determined through their functional groups. The absorption peaks appeared at about 3400 cm^−1^ of all of the samples, which were O-H stretching vibration peaks. The characteristic absorption peak near 2900 cm^−1^ in *β*-glucan and GF (GQ) corresponded to the C-H stretching vibration of sugar. The absorption peaks near 1033 cm^− 1^ were attributed to the stretching vibrations of the CH groups on the benzene ring. Notably, the COO- antisymmetric stretching vibration and the C=C bond on the aromatic ring vibration peak near 1500 cm^− 1^ indicated the existence of phenolic substances. Compared to the individual *β*-glucan and ferulic acid (quercetin) and their physical mixture, the GF (GQ) displayed different degrees of absorption occurring at 3500–1000 cm^−1^ in Figure 3d and Figure 4d, particularly near 3500 cm^−1^, 2800 cm^−1^, 2400 cm^−1^, 1600 cm^−1^, and 1100 cm^−1^. Taking GF as an instance, the new absorption peaks appeared at about 3528 cm^−1^, 2862 cm^−1^, and 2405 cm^−1^. At 1700–1500 cm^−1^, several phenol characteristic absorption peaks appear to merge, forming a new absorption peak of 1672 cm^−1^, which represents the stretching vibration characteristics of C=O and C=C. On the contrary, the absorption peaks in range of 3100–3000 cm^−1^, 1625–1375 cm^−1^, and 900–680 cm^−1^ showed that aromatic ring vibrations all disappeared. These significant changes indicate covalent crosslink existing between *β*-glucan and ferulic acid (quercetin), rather than only simple hydrogen bonding.

### 3.2. Effect of Different One-Way Factors on the Preparation of GF and GQ

The above complexes’ characteristics demonstrate that the equilibrium dialysis method used in this paper can be successfully used to prepare polysaccharide and phenol complexes. Because equilibrium dialysis is a convenient and easily scaled-up method used in mass production, we need to investigate the factors that affect its efficiency and then optimize the parameters. Therefore, we attempted to test the effect of variations in pH, temperature, and PBS concentration on the yield of GF and GQ. The adsorption capacity (calculated using the formula in Section 2.3) of *β*-glucan on ferulic acid or quercetin is used as an indicator to measure the yield of GF and GQ.

**Figure 5:**
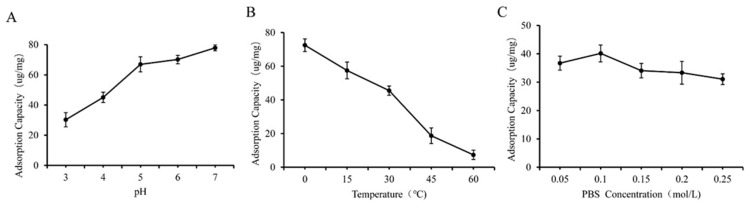
Effect of different factors on the yield of GF. (**A**) pH, (**B**) temperature, (**C**) PBS concentration.

**Figure 6:**
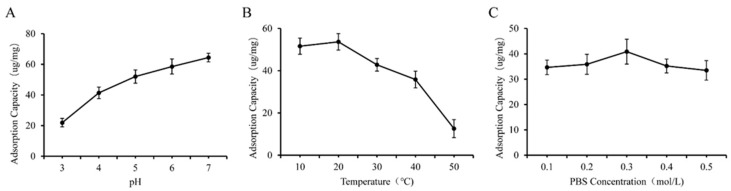
Effect of different factors on the yield of GQ. (**A**) pH, (**B**) temperature, (**C**) PBS concentration.

As shown in Figure 5A and Figure 6A, we observed that higher yields of GF and GQ were acquired at weak acidic or neutral pH compared to strongly acidic pH conditions. The adsorption capacity of *β*-glucan on ferulic acid (quercetin) reached a maximum of 70.12 ug/mg (64.43 ug/mg) at pH 6 (7). Notably, GF and GQ yields were maximized at lower temperatures (0–10 °C) and decreased progressively with increasing temperature (Figure 5B and Figure 6B). These results indicate that the effect of PBS concentration on the yield of the composite was smaller than that of the two factors above. The interaction between *β*-glucan and ferulic acid or quercetin slowly weakens with increasing ionic strength, indicating weak hydrophobic interaction. This result also corresponds to the FTIR results mentioned earlier, indicating that covalent crosslinking played a major role in the combination of *β*-glucan and ferulic acid (quercetin).

### 3.3. Response Surface Optimization

Box–Behnken 7.0 software was used for the analysis of variance (ANOVA) and to model significance based on the data in Table 3 and Table 4, resulting in the following regression model equations: Y_1_ (GF yield) = 72.61 + 29.51 × A − 5.45 × B − 7.34 × C + 1.02 × AB + 0.27 × AC + 0.14 × BC + 15.15 × A^2^ − 8.63 × B^2^ − 8.20 × C^2^; Y_2_ (GQ yield) = 62.976 + 2.51375 × D − 0.1225 × E + 1.17125 × F + 0.0025 × DE − 1.06 × DF − 1.9825 × EF − 5.678 × D^2^ − 7.6905 × E^2^ − 8.153 × F^2^. As shown in Table 5 and Table 6, the F-values of the models were all significant (*p* < 0.0005) and out-of-fit terms (*p* > 0.05), indicating a good fit. The correlation coefficient R^2^ between the response value and the independent variables was 0.9416 (GF) and 0.9918 (GQ), and the adjusted coefficient Adj R^2^ were all greater than 0.9, indicating a high degree of fit between the mathematical model and the actual experimental results. ANOVA results (F-values) revealed the following factor hierarchy for GF/GQ yield: pH > temperature > PBS concentration. pH demonstrated the most significant effect (*p* < 0.01) in the response surface analysis. And, the impact of PBS concentration on the GQ yield model is not significant. Hence, this shows that pH is an important influencing factor.

### 3.4. Effect of Factor Interactions on GF and GQ and Optimal Process Validation

**Figure 7:**
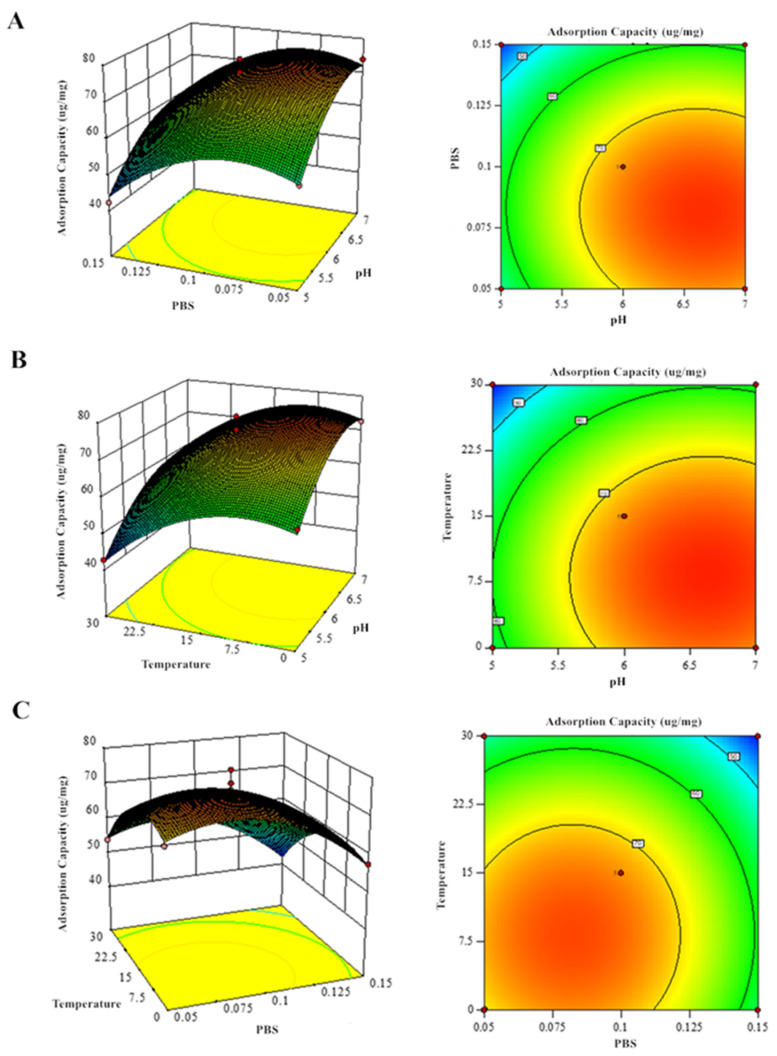
Response surface analysis of GF production showing binary factor interactions: (**A**) PBS–pH, (**B**) temperature–pH, and (**C**) temperature–PBS concentration effects.

**Figure 8:**
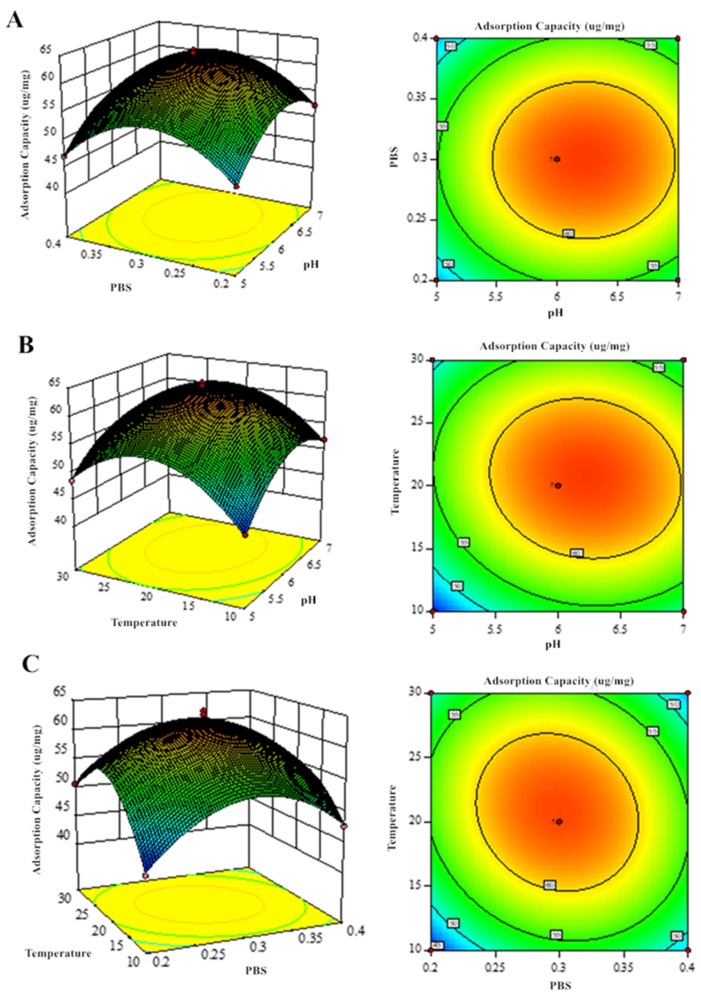
Response surface analysis of GQ production showing binary factor interactions: (**A**) PBS–pH, (**B**) temperature–pH, and (**C**) temperature–PBS concentration effects.

Response surface methodology analysis of the regression equations evaluated how pH, PBS concentration, and temperature influenced GF and GQ yields (Figure 7 and Figure 8). The significant interaction effects (*p* < 0.05) among these factors were confirmed by both the elliptical contour patterns and steep response surface gradients, consistent with ANOVA findings. Eventually, the predicted optimal parameter preparation process for GF (GQ) was determined as follows: a pH of 6.629 (6.216), a final PBS concentration of 0.081 mol/L (0.298 mol/L), and a temperature of 8.196 °C (20.601 °C). The model predicted a yield of 78.02 ug/mg (63.28 ug/mg) of GF (GQ). To simplify experimental execution, the optimal conditions were set at a pH of 6 (6), 0.08 mol/L (0.30 mol/L) of PBS, and 8 °C (20 °C), yielding 76.33 ug/mg (61.27 ug/mg) of GF (GQ). The negligible deviation between predicted and actual results confirms the robustness of the model-derived parameters, highlighting its accuracy in identifying critical extraction factors.

### 3.5. Antioxidant Activity In Vitro

**Figure 9:**
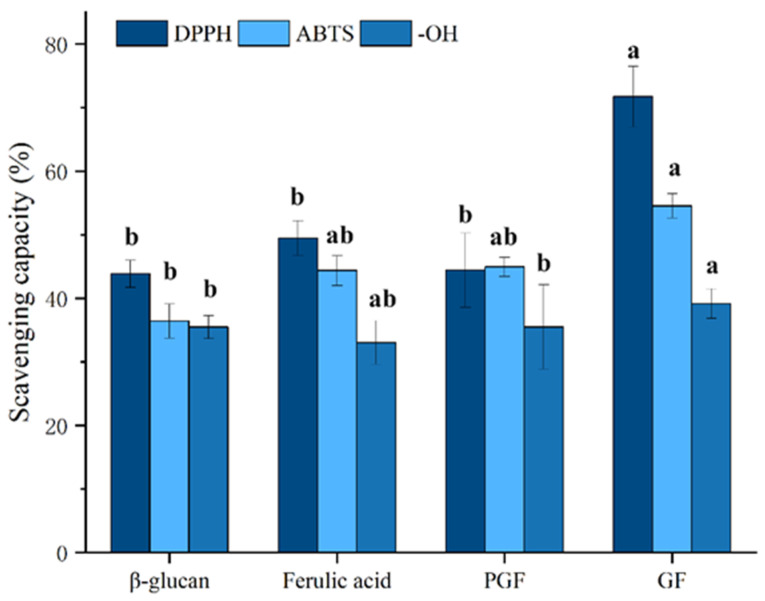
The in vitro antioxidant capacities of GF. Data are expressed as mean ± SEM, and different letters indicate significant differences in the same category (*p* < 0.05).

**Figure 10:**
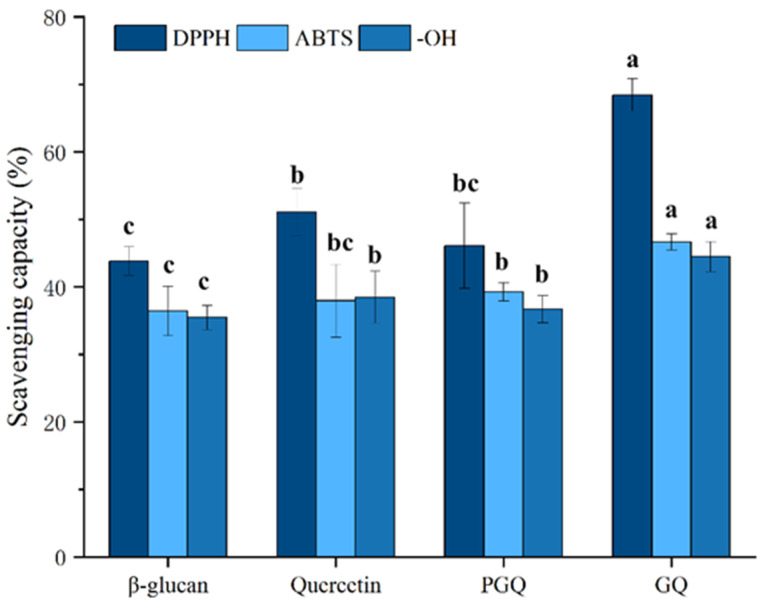
The in vitro antioxidant capacities of GQ. Data are expressed as mean ± SEM, and different letters indicate significant differences in the same category (*p* < 0.05).

Figure 9 demonstrates a concentration-dependent antioxidant response across all sample groups. At the same concentration, the DPPH radical scavenging capacity was ranked as follows: GF (71.74 ± 4.7%) > ferulic acid (49.50 ± 2.7%) > PGF (44.43 ± 5.8%) > *β*-glucan (43.84 ± 2.1%). Similar trends were observed for ABTS radical scavenging (GF: 54.56 ± 1.9%; ferulic acid: 44.37 ± 2.3%; PGF: 44.95 ± 1.5%; *β*-glucan: 36.42 ± 2.7%) and hydroxyl radical elimination (GF: 39.16 ± 2.3%; ferulic acid: 33.06 ± 3.4%; PGF: 35.51 ± 6.6%; *β*-glucan: 35.47 ± 1.8%). Different antioxidant activity tests have shown that polysaccharide–phenol conjugation significantly (*p* < 0.05) enhances antioxidant capacity compared to individual components and physical mixtures of the two compounds, which has also been validated on *β*-glucan–quercetin complexes (Figure 10) (DPPH radical scavenging capacity: GQ (68.44 ± 2.4%), quercetin (51.08 ± 3.4%), PGQ (46.10 ± 6.3%), *β*-glucan (43.84 ± 2.1%); ABTS radical scavenging capacity: GQ (46.67 ± 1.2%), quercetin (37.96 ± 5.4%), PGQ (39.29 ± 1.4%), *β*-glucan (36.42 ± 2.7%); hydroxyl radical elimination: GQ (44.49 ± 2.2%), quercetin (38.48 ± 3.8%), PGQ (36.72 ± 2.0%), *β*-glucan (35.47 ± 1.8%)). However, through statistical analysis, we found that PGF and PGQ showed almost equivalent antioxidant activity with individual ferulic and glucan on DPPH, ABTS, and OH^−^ assay. Although there may be intuitive highs and lows on the bar chart, they have not reached a significant difference (Figure 9 and Figure 10).

## 4. Discussion

Polysaccharides and phenols are the main characteristic active substances in highland barley, which exhibit significant physiological activities in regulating lipid metabolism and enhancing antioxidant capacity. Previous studies have shown that barley polysaccharide can indirectly affect lipid metabolism by improving gut microbiota, while the phenol components effectively reduce vascular oxidative stress through antioxidant and anti-inflammatory effects, thereby improving lipid metabolism [22,23,24]. Given these multifaceted advantages, polysaccharide–phenol conjugates hold strong promise for applications in functional food development. However, in practical applications, due to limitations in solubility and bioavailability, the effect of a single component may not be significant enough. In recent years, studies have found that polysaccharide and phenol can form complexes through non-covalent or covalent bonds, enhancing their stability and bioavailability in vivo. For example, the tea phenol can bind with oat *β*-glucan through strong hydrogen bonds, forming a complex that not only enhances the stability of phenol but also increases antioxidant activity, exhibiting especially high superoxide dismutase and glutathione peroxidase activity in the liver [25]. These studies indicate that PPC have significant potential in enhancing biological activity. However, there is still limited research on the structural characteristics, binding mechanisms, and biological functions of highland barley PPC [8,9]. In this study, we prepared two complexes of GF and GQ that may exist in highland barley using the equilibrium dialysis method to further study the binding mode, morphological characteristics, and antioxidant activity of the complexes. Membrane dialysis is convenient and scalable for industrial production technology, which has a wide range of applications in the food industry for the filtration, concentration, and fractionation of dairy products, fruit and vegetable juice, and beer [26]. The beer industry employs sequential membrane processes for product refinement. Microfiltration recovers beer from surplus yeast, followed by reverse osmosis for alcohol reduction. Final clarification is achieved through plate-and-frame crossflow microfiltration, which effectively removes residual bacteria and spores while preserving product quality. Given the mature application of membrane technology, the use of dialysis membranes in mild environments to prepare barley PPC, as in this article, may be easier to scale up and apply in future industrial production compared to chemical grafting reactions.

Phenol–polysaccharide interactions occur through two distinct mechanisms: covalent and non-covalent bonding [7]. Current research indicates that common polysaccharides (including cyclodextrins, glucans, chitosan, pectin, and cellulose) are primarily associated with phenol via non-covalent interactions [27]. Non-covalent PPC primarily develop through reversible molecular interactions, including hydrogen bonds, ionic attractions, van der Waals forces, and hydrophobic effects. In contrast, covalent conjugation between these biomolecules occurs either spontaneously or through catalysis by enzymes, pH modifiers, or metal ions [28]. The principal covalent binding mechanisms involve enzymatic oxidation, carbodiimide-mediated crosslinking, and radical-induced coupling. In this study, the complexes GF and GQ showed significant differences in their infrared spectral characteristics with the disappearance and addition of characteristic absorption peaks compared with individual *β*-glucan and ferulic acid (quercetin) and their physical mixture. In addition, the microstructure morphology of two complexes shows it forming a dense new structure, indicating the existence of covalent chemical bonds in crosslinking between *β*-glucan and ferulic acid (quercetin). Previous research indicates that hydroxyl radicals produced during phenol–polysaccharide redox reactions can target vulnerable functional groups in polysaccharide, generating polysaccharide macromolecular radicals [29]. These reactive intermediates facilitate subsequent redox processes with phenol, ultimately promoting covalent bond formation between the two components. Diao et al. synthesized quercetin–chitosan conjugates through radical-mediated grafting, with UV and FTIR spectroscopy verifying successful covalent modification [30]. They found that the appearance of two characteristic absorption bands at 1630 cm^−1^ and 1406 cm^−1^ confirmed the presence of quercetin molecules in the conjugate. Additionally, the persistence of the C–O–C bridge vibration at 1151 cm^−1^ in the quercetin–chitosan spectrum verified the preservation of the chitosan backbone structure, collectively demonstrating successful quercetin–chitosan formation. In our study, at 1672 cm^−1^ and 1654 cm^−1^, a new merging peak appeared through FTIR spectroscopy of GF and GQ, respectively. And, at a wavenumber of 1600–1200 cm^−1^, the typical bands for the ferulic acid and the quercetin, which are due to the stretching vibration of its benzoyl rings, disappeared instead. The asymmetric stretching vibration of the C–O–C bridge of *β*-glucan, characterized by the band at 1100–1000 cm^−1^, still appeared in the GF and GQ spectrum, revealing that the skeleton structure of polysaccharide chains remains in complexes. These significant changes in absorption peaks indicate the interaction between *β*-glucan and ferulic acid (quercetin) crosslinking with the covalent bond between them.

Systematic examination of intrinsic and extrinsic factors governing phenol–polysaccharide binding facilitates the rational design of synergistic complexes. Such studies establish fundamental principles for maximizing bioactivity. Exogenous factors include pH, temperature, and ionic concentration [8]. The pH level significantly influences the binding behavior between phenol and polysaccharide. Research demonstrates pH-dependent variations in phenol–polysaccharide interactions. Lin et al. [31] reported optimal anthocyanin–pectin binding at pH 3.6 (range: 2.0–4.5), with quinoid anthocyanin structures showing enhanced polysaccharide affinity. Wu et al. [15] identified pH 6.0 as optimal for tea phenol–*β*-glucan complexes, while ferulic acid shows increased polysaccharide binding with pH elevation, an effect less pronounced for catechins. Temperature is another important factor. The temperature’s effects on phenol–polysaccharide interactions were demonstrated by Fernandes et al. [32], showing that elevated temperatures disrupt glucosidic linkages and weaken pectin–anthocyanin associations by interfering with electrostatic and hydrogen bonding. Similarly, Wu et al. [15] observed a substantial decrease in tea phenol-*β*-glucan binding affinity when temperature increasing from 20 °C to 60 °C, suggesting hydrogen bonding’s crucial role in these molecular interactions. Ionic characteristics also significantly influence phenol–polysaccharide binding interactions. Peng et al. [33] determined that optimal binding between gallic acid and lotus root polysaccharide occurred at 20 mmol/L of NaCl, whereas epigallocatechin showed minimal binding at this concentration. Phan et al. [6] reported that *β*-glucan–tea phenol interactions remained stable below 100 mmol/L of NaCl but were significantly inhibited at 0.5 mol/L, demonstrating NaCl’s concentration-dependent modulation of hydrophobic interactions. Studies reveal that ionic conditions influence phenol–polysaccharide binding primarily through polysaccharide conformational changes and the suppression of hydrophobic interactions.

In this study, the factors pH, PBS concentration, and temperature were investigated to see how they affect the constitution of GF and GQ, with pH having the greatest effect. Then, through response surface methodology, the optimal conditions for GF and GQ were identified. The optimal conditions for GF and GQ were pH 6.5, 0.08 mol/L PBS, and 8 °C and pH 6, 0.3 mol/L PBS, and 20 °C, respectively. The results are consistent with some reports that weakly acidic and neutral environments (pH 6–7) are more conducive to the formation of complexes. The above pH values are similar to the acid–base environment of the human oral and intestinal tract, which provides favorable conditions for the formation and stability of complexes between *β*-glucan and ferulic acid (quercetin) in the oral and intestinal systems, further exerting positive effects, such as antioxidants and the regulation of gut microbiota. It was found that the combination of polysaccharides and phenols can play the role of a phenol carrier, which provides important support for the application of phenols in biomedicine. For example, the complex exhibited a good swelling rate and in vivo slow-release properties, which contributed to it conjugating phenol drugs and achieving the effect of controlling drug release. Chatterjee et al. [34] prepared the chitosan–ferulic acid microcapsules through free radical grafting. The results indicate that the microcapsules showed good controllable release ability and anti-inflammatory effect. Hence, on the contrary, the stability of the complex in a strongly acidic environment in the stomach deserves further investigation, which may directly determine whether the complex can enter the intestine stably.

In addition, lower temperature and ion concentrations are also beneficial for promoting *β*-glucan and ferulic acid (quercetin) monomer recombination, which proves that intermolecular forces, including hydrogen bonding and hydrophobic interactions, exist in binding modes between phenol–polysaccharide complexes. Although research on the impact of the reaction environment on complex formation is currently detailed, the mechanism through which polysaccharides’ and phenols’ own structures and reaction activities affect complex formation remains the focus of further research. In addition, the optimal preparation conditions for GF and GQ given in this article were aligned with industrial feasibility. Neutral and low ion concentration reaction conditions are easy to achieve and cost-effective for the producer. In addition to the low-temperature (8 °C) reaction conditions required for GF production, which may increase energy costs, the temperature conditions for producing GQ are close to room temperature (20 °C), which is within a controllable range.

Oxidative stress results from free radical overproduction that overwhelms endogenous antioxidant defenses, leading to cellular damage. Research indicates that oxidative stress, driven by excessive free radicals, is a primary contributor to several diseases [35,36]. Antioxidant capacity, which reflects free radical scavenging ability, serves as a key metric for evaluating this protective effect. In our study, the antioxidant activity of the complexes was evaluated. Notably, comparative evaluation showed that the complexes GF and GQ consistently surpassed the polysaccharide or phenol monomer compound and their mixture in DPPH, ABTS, and hydroxyl radical scavenging capacity. The observed high free radical scavenging ability of the complex may be mainly attributed to the changes in the physical properties of polysaccharides due to polyphenols, such as solubility and emulsifying ability. For example, researchers have revealed through crystallographic analysis that quercetin grafting disrupts chitosan’s crystalline packing, increasing molecular accessibility [34]. This structural loosening exposes additional hydrogen-donating sites, accounting for the quercetin–chitosan conjugate’s significantly elevated ABTS^+^ and DPPH∙ scavenging activity compared to precursors. As another example, the addition of polyphenols can change the structure of polysaccharides, forming a denser structure, thereby avoiding the formation of larger aggregates through the steric hindrance effect and improving the antioxidant capacity of the complex. Therefore, we believe that using simple processing methods to promote the formation of phenol–polysaccharide complexes in highland barley is an effective approach to enhancing its antioxidant activity, which may provide theoretical and technical support for the high-value utilization and processing of highland barley. However, we still need to recognize the possibility of a gap between the free radical scavenging ability in vitro and the actual antioxidant activity in vivo. The DPPH, ABTS, and hydroxyl radical scavenging experiment was conducted in a non-biological system and did not involve complex biological metabolic processes or enzyme systems. Therefore, its results mainly reflect the direct chemical reaction ability of GF and GQ. The antioxidant activity of the complexes in vivo is influenced by various factors, such as digestion, metabolism, bioavailability, and the antioxidant system, in the biological environment. Therefore, accurate evaluation of the antioxidant levels of GF and GQ is necessary for future research.

## 5. Conclusions

GF and GQ, two phenol–polysaccharide complexes with covalent and non-covalent bonds, were prepared from highland barley via the equilibrium dialysis method. They showed significant differences in their infrared spectral characteristics, microstructure morphology, and antioxidant activity compared with individual *β*-glucan and ferulic acid (quercetin) and their physical mixture. Experimental results demonstrated that three key parameters, including pH, reaction temperature, and phosphate buffer concentration, significantly influenced the yield of the complexes. We also determined the optimal method for preparing the two complexes based on the three factors above through a response surface methodology. However, future research should still pay special attention to the following aspects: 1. analyzing the structure–function correlations in both native and engineered PPC from highland barley; 2. using NMR and LC/MS to analyze the precise structure of GF and GQ; 3. further evaluate the antioxidant capacity of the complex in vivo; and 4. considering the potential application of the complexes in functional foods in the future, evaluate the toxicity and shelf stability of GF and GQ, as well as other necessary assessments for food. Above all, the GF and GQ complexes show significant potential as novel bioactive ingredients for functional foods and pharmaceutical applications.

## Figures and Tables

**Table 1:** Experimental design of response surface analysis for GF.

No.	Factor	Level		
		−1	0	1
A	pH	5	6	7
B	PBS (mol/L)	0.05	0.1	0.15
C	Temperature (°C)	0	15	30

**Table 2:** Experimental design of response surface analysis for GQ.

No.	Factor	Level		
		−1	0	1
D	pH	5	6	7
E	PBS (mol/L)	0.2	0.3	0.4
F	Temperature (°C)	10	20	30

**Table 3:** Response surface experimental design and results for GF.

Number	pH	PBS Concentration (mol/L)	Temperature (°C)	Y: Adsorption Capacity (ug/mg)
	A	B	C	
1	7	0.1	0	72.68
2	7	0.15	15	58.96
3	6	0.15	30	43.24
4	5	0.05	15	55.14
5	6	0.1	15	74.23
6	5	0.15	15	42.24
7	6	0.05	30	53.87
8	5	0.1	30	52.87
9	7	0.05	15	73.95
10	6	0.1	15	77.86
11	6	0.15	0	57.41
12	5	0.1	0	59.05
13	7	0.1	30	57.41
14	6	0.1	15	72.5
15	6	0.1	15	68.32
16	6	0.1	15	70.13
17	6	0.05	0	68.59

**Table 4:** Response surface experimental design and results for GQ.

Number	pH	PBS Concentration (mol/L)	Temperature (°C)	Y: Adsorption Capacity (ug/mg)
	D	E	F	
1	7	0.3	30	51.55
2	6	0.4	10	47.59
3	6	0.3	20	64.46
4	6	0.3	20	62.82
5	5	0.2	20	47.38
6	6	0.3	20	62.17
7	5	0.3	10	44.6
8	7	0.4	20	51.8
9	6	0.3	20	61.52
10	7	0.2	20	52.3
11	5	0.4	20	46.9
12	6	0.2	10	43.7
13	7	0.3	10	51.96
14	6	0.2	30	50.6
15	6	0.4	30	46.6
16	6	0.3	20	63.9
17	5	0.3	30	48.5

**Table 5:** Regression analysis of yield model and regression coefficients for GF.

Source	Sum of Squared Deviations	Degrees of Freedom (df)	Mean Square	F-Value	*p*-Value	Significance
Model	2130.51	9	236.72	24.75	0.0002	**
A	507.21	1	507.21	53.02	0.0002	**
B	308.76	1	308.76	32.28	0.0007	**
C	455.11	1	455.11	47.57	0.0002	**
AB	1.09	1	1.09	0.1142	0.7454	ns
AC	0.2070	1	0.2070	0.0216	0.8872	ns
BC	0.0756	1	0.0756	0.0079	0.9316	ns
A^2^	172.75	1	172.75	18.06	0.0038	**
B^2^	313.61	1	313.61	32.78	0.0007	**
C^2^	283.13	1	283.13	29.60	0.0010	**
Residual	66.96	7	9.57			
Misfit term	12.21	3	4.07	0.2974	0.8266	ns
Pure error	54.75	4	13.69			
Total	2197.47	16				
R^2^ = 0.9416, Adj R^2^ = 0.9303, PredR^2^ = 0.8722						

Significance levels were defined as follows: ** *p* < 0.01 (highly significant) and ns (not significant) for *p* > 0.05.

**Table 6:** Regression analysis of yield model and regression coefficients for GQ.

Source	Sum of Squared Deviations	Degrees of Freedom (df)	Mean Square	F-Value	*p*-Value	Significance
Model	822.26	9	91.36	93.67	<0.0001	**
D	50.55	1	50.55	51.83	0.0002	**
E	0.1200	1	0.1200	0.1231	0.7360	ns
F	10.97	1	10.97	11.25	0.0122	*
DE	0.0000	1	0.0000	0.0000	0.9961	ns
DF	4.49	1	4.49	4.61	0.0690	ns
EF	15.72	1	15.72	16.12	0.0051	**
D^2^	135.75	1	135.75	139.18	<0.0001	**
E^2^	249.03	1	249.03	255.32	<0.0001	**
F^2^	279.88	1	279.88	286.96	<0.0001	**
Residual	6.83	7	0.9753			
Misfit term	0.9588	3	0.3196	0.2178	0.8795	ns
Pure error	5.87	4	1.47			
Total	829.09	16				
R^2^ = 0.9918, Adj R^2^ = 0.9812, PredR^2^ = 0.9704						

Significance levels were defined as follows: ** *p* < 0.01 (highly significant), * *p* < 0.05 (significant), and ns (not significant) for *p* > 0.05.

## Data Availability

The original contributions presented in this study are included in the article. Further inquiries can be directed to the corresponding author.
